# Digital Developments in Society That Persons 75 Years and Older Have Been Part of: A Scoping Review

**DOI:** 10.1093/geroni/igab046.993

**Published:** 2021-12-17

**Authors:** Moonika Raja, Jorunn Bjerkan, Ingjerd Kymre, Kathleen Galvin, Lisbeth Uhrenfeldt

**Affiliations:** 1 Nord University, Nord University, Bodø, Nordland, Norway; 2 Nord University, Nord University, Levanger, Nord-Trondelag, Norway; 3 University of Brighton, University of Brighton, England, United Kingdom

## Abstract

The population in Europe is ageing and people are becoming more than ever dependent on digital technologies. The present study aims to map relevant evidence about digital developments in society involving people aged 75 and over in European countries. It focuses on their experiences and the main barriers to, and facilitators of, societal digital demands. Scoping reviews can be used when the purpose is to identify types of available evidence and clarify concepts, this process was guided by a framework proposed by Arksey and O`Malley. The studies included in the review covered digital technology, digital devices and telehealth, and the context covered participants` own home or surroundings. A comprehensive search was made on CINAHL, Embase, Pubmed/MEDLINE, Scopus and Open Grey. Out of 727 identified citations, 13 sources which met the inclusion criteria (9 original study articles, 2 theses, 1 letter about a product and 1 project report). The studies included varied in their focus, design and location. Older European citizens have experienced technology making life easier and the opposite. The outstanding facilitator found was that technology should be easy to use. Interestingly, both social support and lack of social support were found as facilitators of using new technology and difficulty in remembering the instructions was seen as an important barrier. As technology develops rapidly, there is a need for new and additional research among older European citizens. Future research should cover participants` access to the devices, social support and the technical solutions most relevant to older people today.

